# Supplementary material for: Clinical Manifestations, Macrolide Resistance, and Treatment Utilization Trends of Mycoplasma pneumoniae Pneumonia in Children and Adolescents in South Korea
Source: Microorganisms. 2024 Aug 31;12(9):1806. doi: 10.3390/microorganisms12091806 (PMC11434231; doi:10.3390/microorganisms12091806)
Supplement: Supplementary file 1 [file microorganisms-12-01806-s001.zip › MP_supplementary table-2024-08-04.pdf]

Supplementary Table S1. Brand of PCR kits and the antibody test methods used for diagnosis in each hospital.

| Institution | PCR kits | Antibody test |
|---|---|---|
| CHA Bundang Medical Center | BIOFIRE® Respiratory 2.1 Panel (bioMérieux, Marcy-l'Étoile, France) | M. pneumoniae IgG/M (EONE, Incheon, Korea) |
| Samsung Medical Center | M. pneumoniae PCR (SCL, Yongin, Korea)<br>BIOFIRE® Respiratory 2.1 Panel (bioMérieux, Marcy-l'Étoile, France) | Serodia-Myco II (Fujirebio, Tokyo, Japan) |
| Seoul St. Mary's Hospital | BioCore M. pneumoniae Real-Time PCR Kit (Invites BioCore, Seoul, Korea) | LIAISON® M. pneumoniae IgG (Diasorin, Saluggia, Italy)<br>Chorus Mycoplasma IgM (DIESSE, Monteriggioni, Italy) |
| Inha University Hospital | Allplex PneumoBacter Assay (Seegene, Seoul, Korea) | Serodia-Myco II (Fujirebio, Tokyo, Japan) |
| Nowon Eulji Medical Center | Bacterial Multiplex PCR (GCLabs, Yongin, Korea) | Chorus Mycoplasma IgG/M (DIESSE, Monteriggioni, Italy) |
| Seoul National University Children’s Hospital | Ecoli Dx (Amplisens, Prague, Czech Republic) | Serodia-Myco II (Fujirebio, Tokyo, Japan) |
| Korea University Anam Hospital | M. pneumoniae PCR (GCLabs, Yongin, Korea) | N/A |
| Seoul National University Bundang Hospital | NxTAG RPP (Luminex Corporation, Austin, USA) | R-FIND M. pneumoniae IgG/M (SG Medical, Seoul, Korea) |
| Chungnam National University Hospital | BIOFIRE® Respiratory 2.1 Panel (bioMérieux, Marcy-l'Étoile, France)<br>Allplex PneumoBacter Assay (Seegene, Seoul, Korea) | LIAISON® M. pneumoniae IgG/M (Diasorin, Saluggia, Italy) |
| Hallym University Kangnam Sacred Heart Hospital | Allplex PneumoBacter Assay (Seegene, Seoul, Korea) | LIAISON® M. pneumoniae IgG/M (Diasorin, Saluggia, Italy) |
| Pusan National University Yangsan Hospital | Bacterial Multiplex PCR (SCL, Yongin, Korea)<br>M. pneumoniae PCR (Seegene, Seoul, Korea)<br>BIOFIRE ® Respiratory 2.1 Panel (bioMérieux, Marcy-l'Étoile, France) | LIAISON® M. pneumoniae IgG/M (Diasorin, Saluggia, Italy) |
| Chungbuk National University Hospital | Allplex PneumoBacter Assay (Seegene, Seoul, Korea)<br>M. pneumoniae PCR (SCL, Yongin, Korea) | LIAISON® M. pneumoniae IgG/M (Diasorin, Saluggia, Italy) |
| Jeju National University Hospital | Bacterial Multiplex PCR (EONE, Incheon, Korea) | N/A |

N/A, not available.
