# Supplementary figures and images for: Clinical Manifestations, Macrolide Resistance, and Treatment Utilization Trends of Mycoplasma pneumoniae Pneumonia in Children and Adolescents in South Korea
Source: Microorganisms. 2024 Aug 31;12(9):1806. doi: 10.3390/microorganisms12091806 (PMC11434231; doi:10.3390/microorganisms12091806)

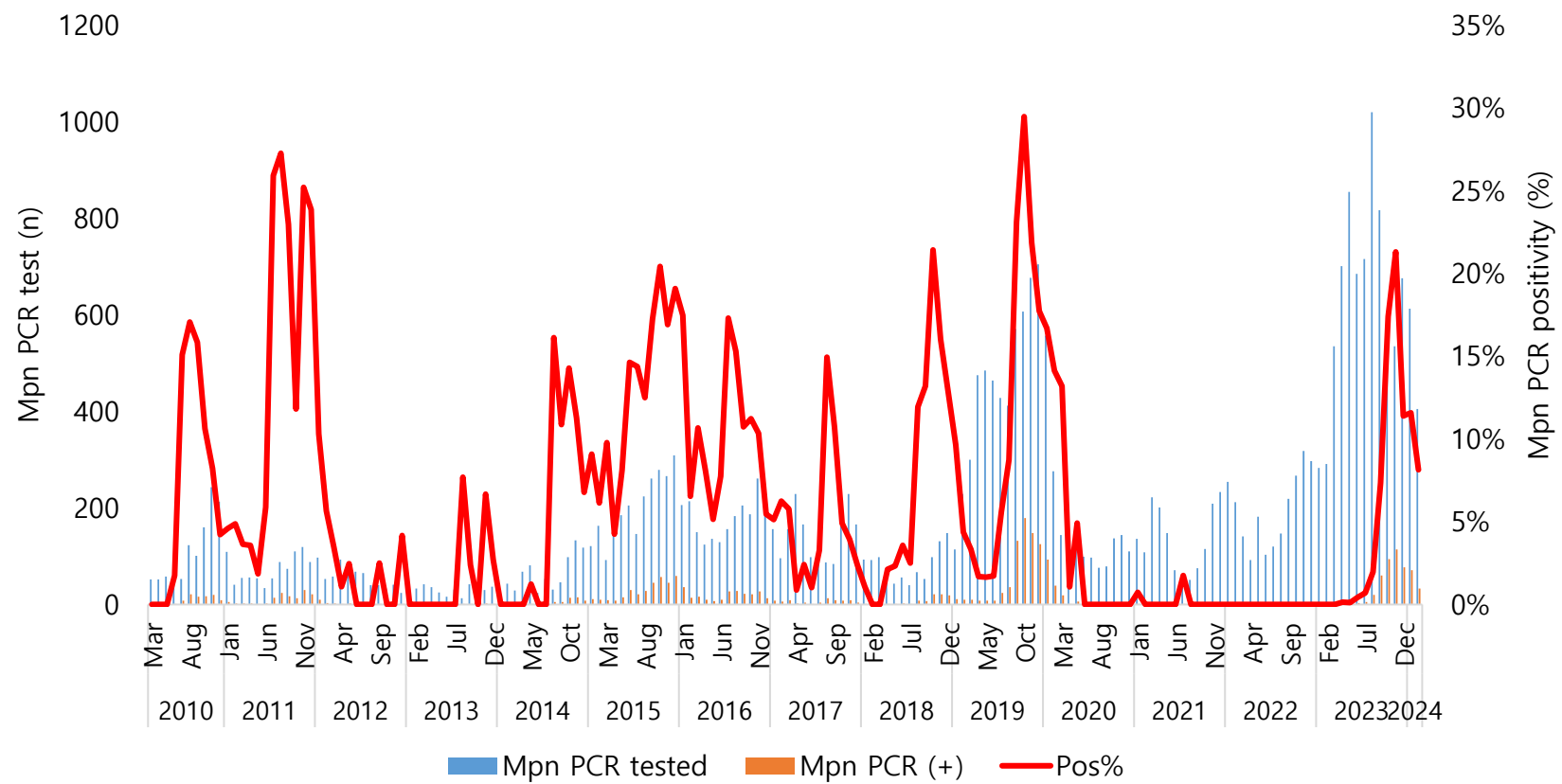

Suppl. Fig. S1

Supplement: Supplementary file 1 [file microorganisms-12-01806-s001.zip › Suppl Fig-2024-08-04.pdf]
